# On the Treatment of Whooping-Cough by Belladonna and Sulphate of Zinc

**Published:** 1865-01

**Authors:** E. Garraway


### *On the Treatment of Whooping-Cough by Belladonna and Sulphate of Zinc.* By E. Garraway, Esq., M. R. C. S. E.

Until a comparatively recent period the treatment of whooping-cough may justly have been regarded as one of the *approbia medici.* The authorities of the profession not even yet being agreed upon its true pathology, it is no marvel that the treatment has been more or less empirical; that notwithstanding there have been countless "infallible specifics," yet the ordinary duration of whooping-cough, except when the epidemic prevails in a mild form, is still three, and even during the winter as much as six, months.

I am not about to enter into the question of the specific nature of the disorder, whether its seat be in the mucous or muscular lining of the air-passages, in the brain, the spinal cord, the stomach, the pneumogastric nerve or elsewhere. The preponderance of opinion in the present day is largely in favor of its being a nervous disorder, and at least it would appear to have quite as much claim to be so considered as asthma, ague, chorea, epilepsy or other convulsive disorders which it has been found impossible to localize. In mild, uncomplicated cases of whooping-cough, not even functional derangement of any organ can be detected. In the intervals of the attacks of cough and concomitant spasm, a state of perfect health subsists.

The observations I have to offer are solely confined to "treatment," and one form of treatment—viz: that by belladonna and sulphate of zinc. A very extensive prevalence of whooping-cough during the last winter has afforded considerable opportunities of testing the value of these remedies; indeed, I have treated every one of my cases—numbering between fifty and sixty, and these limited to private practice—with zinc and belladonna, to the exclusion of all other remedies. Of course the supervension of anything like bronchitis or pulmonary congestion has required the administration of emetics; but these cases have been rare, and in only two have I had occasion to suspend the belladonna treatment for two or three days, and substitute for it ipecacuanha, antimony, and external counter-irritants. In the comparatively small number of fifty cases, it is not surprising that I have no death to record. The mortality, however, has been exceedingly large—in London amounting to from 80 to 100 a week during several months—once rising to 124. In my own district, the registrar informs one that, in a population of 9000, he recorded from October to April fourteen deaths as due to whooping-cough.

The first three cases I saw occurred in a school. They were boys between the ages of six and nine. The paroxysms in all were moderately frequent and severe; the general health was good. Two were placed under my care; the third was left to take his chance. In eighteen days my patients had entirely ceased to cough, whilst the other boy was in precisely the same state as when I first saw him, and so continued for several weeks afterwards.

A little girl between two and three years old was brought to me, whose fits were described to be of the most violent character; and indeed the poor child's appearance did not belie the mother's statement, for both conjunctivæ were so entirely ecchymosed that the white sclerotic coat was invisible: no blow, however severe, could have produced a more complete pair of black eyes. For a fortnight there was not the slightest improvement, and I was in consequence much troubled to keep my patient up to her medicine; in one week more, however, the cough was gone, and the ecchymosis had completely disappeared.

Three weeks afterwards the mother came for the same medicine for her infant, eight months old, who coughed and whooped, she told me, a dozen or twenty times in the day and some half dozen times in the night—not a severe case. I exhorted her not to be disheartened for three weeks, but to come regularly for her supplies. She never made her appearance again; and on meeting her some time afterwards, and expressing my regret that the treatment had not been followed up, she replied, "Why, the medicine you gave me lasted a week, and by that time the cough had entirely ceased, and has never returned."

These are samples (which might be multiplied) of the more favorable class of cases. Others were marked by a steady improvement, commencing at once and continuing day by day; but at the same time I must admit that not a few required fully three weeks to make a decided impression. After this period, however, the improvement was most rapid; indeed, treatment was seldom continued beyond the third week. The most lengthened period for which, in any case, belladonna was administered was five weeks. I found that if by this time the cough was not quite gone, it was nevertheless desirable to discontinue the remedy; as it would seem that after the system was thoroughly saturated, a further persistence deranged the general health without any commensurate advantage accruing: the tongue became coated, the appetite failed, and vomiting without cough would occasionally occur. On ceasing, however, to take the medicine, not only did all these untoward symptoms subside, but the remains of the cough vanished with them.

Marsh miasmata appear to render whooping-cough, as they do most other forms of disease, more intractable; and two children I could do nothing with until removed from the swamp in which they resided.

The mode of administering the belladonna was in the form of extract, either diffused in water with the sulphate of zinc and sufficient syrup to make it agreeable to young children, or, to those who were old enough and preferred it, in the form of pills—the dose being from one-sixth to one-fourth of a grain of the extract, and one-half to a grain of the zinc, three or four times a day, steadily increasing the amount till, at the end of three weeks, children of five or six years old would be taking from four to six grains of belladonna, and twice that quantity of sulphate of zinc, daily.

So far as my investigation went, it would appear that both the tolerance of the remedy and the speedy subsidence of the disorder were in inverse proportion to the age of the subject—a child of eight or ten weeks old bearing a much larger proportionate dose than one of eight or ten years and manifesting a much more rapid improvement. In this I find I am in accord with other observers.

Dilatation of the pupils and indistinctness of vision commonly came on after a few days. When these effects manifested themselves the dose was diminished somewhat; but having been assured by Dr. Fuller, of St. George's Hospital, whose experience of the physiological effects of belladonna has been considerable, that no permanent injury ever resulted from this condition, I did not think it necessary to interfere with the general line of treatment.

In two cases more decided poisonous symptoms were developed. One was a little girl of six, who had reached the amount of six grains daily, and whose pupils had been dilated more or less for a week. This child became one day, as her parents termed it, "silly," delivered wrong messages, gave inapt answers, asked what had become of her sisters when they were present, and talked in an incoherent and ridiculous manner. This state quite passed off the next day by discontinuing the medicines.

The other case was that of a very delicate little girl of four years, who had attained to four grains a day. I was called to her in the night under the great alarm of her parents. She had been in a state of immoderate mirthfulness and excitement during the evening, and on being put to bed could not be quiet, and at length became delirious, singing, calling for her mamma and nurse, of whose presence she was unconscious, picking at the bed-clothes, seeing imaginary objects—in fine, presenting a train of symptoms very analogous to what we witness in delirium tremens in the adult. This state was succeeded by three or four hours of refreshing sleep, such as the child had not experienced for many nights. On awaking she was perfectly restored, and from that hour the improvement was remarkably rapid. I need hardly say I suspended the remedy for a day, and gave it afterwards in diminished doses.

Such, in brief, has been my experience of the treatment of whooping-cough by belladonna and sulphate of zinc. Which of these two had the greater share in the favorable results I am unable to say, since they were not in any case dissociated; but as similar results have followed the exhibition of belladonna alone in other hands, it is reasonable to conclude that this was the more active agent. It ought perhaps, in fairness, to be recorded that some cases which were cured at the beginning of winter showed a tendency to a return of the disorder when the cold north-east winds of March set in. Few of these cases came under treatment, as the relapse was not of a severe character; when, however, belladonna was given, its effects were marked and immediate.

From the limited data afforded by fifty cases of whooping-cough, it would be presumptuous in me to speak authoritatively as to the propriety of this or any other mode of treatment; nor should I have obtruded these observations were I not impressed with the conviction that the belladonna treatment is not so generally, or at least so universally, accepted as I conceive it deserves; and in this I am borne out by the fact that in one of our best modern works upon the diseases of children—namely, Dr. West's—the very name of belladonna in connection with whooping-cough does not once occur.

